# Destruction of *Staphylococcus aureus* biofilms by combining an antibiotic with subtilisin A or calcium gluconate

**DOI:** 10.1038/s41598-021-85722-4

**Published:** 2021-03-18

**Authors:** JingJing Liu, Jean-Yves Madec, Alain Bousquet-Mélou, Marisa Haenni, Aude A. Ferran

**Affiliations:** 1 Unité Antibiorésistance et Virulence Bactériennes, Université de Lyon - ANSES Laboratoire de Lyon, Lyon, France; 2 INTHERES, INRAE, ENVT, Université de Toulouse, Toulouse, France

**Keywords:** Biofilms, Antibiotics

## Abstract

In *S. aureus* biofilms, bacteria are embedded in a matrix of extracellular polymeric substances (EPS) and are highly tolerant to antimicrobial drugs. We thus sought to identify non-antibiotic substances with broad-spectrum activity able to destroy the EPS matrix and enhance the effect of antibiotics on embedded biofilm bacteria. Among eight substances tested, subtilisin A (0.01 U/mL) and calcium gluconate (CaG, Ca^2+^ 1.25 mmol/L) significantly reduced the biomass of biofilms formed by at least 21/24 *S. aureus* isolates. Confocal laser scanning microscopy confirmed that they both eliminated nearly all the proteins and PNAG from the matrix. By contrast, antibiotics alone had nearly no effect on biofilm biomass and the selected one (oxytetracycline-OTC) could only slightly reduce biofilm bacteria. The combination of OTC with CaG or subtilisin A led to an additive reduction (average of 2 log_10_ CFU/mL) of embedded biofilm bacteria on the isolates susceptible to OTC (MBC < 10 μg/mL, 11/24). Moreover, these two combinations led to a reduction of the embedded biofilm bacteria higher than 3 log_10_ CFU/mL for 20–25% of the isolates. Further studies are now required to better understand the factors that cause the biofilm produced by specific isolates (20–25%) to be susceptible to the combinations.

## Introduction

Bacterial biofilms are surface-associated multicellular communities in which bacteria are embedded in a self-produced extracellular polymeric substance (EPS), a matrix mostly composed of exopolysaccharides and proteins^1^. Owing to their recalcitrance to antibiotic treatments and to immune host defences, biofilm-associated infections are often chronic and a cause of frequent relapses^2^. Such infections have been reported to be involved in 65% to 80% of all human bacterial infections^3^ and give rise to major issues in human and veterinary medicine^4,5^. The high prevalence of persister cells in biofilms as well as the complex chemical composition and structure of the extracellular matrix have been described as the key critical factors responsible for the very low activity of antimicrobial drugs on biofilms^5–8^.

The strategies to manage *S. aureus* biofilms include early physical removal of colonised materials or local delivery of high doses of antibacterial chemotherapy^9^ when possible. However, even after therapy with very high concentrations of antibiotics for several weeks, the clinical cure rate of *S. aureus* infections remains low^10^. Indeed, previous studies suggested that the EPS matrix that remains after the bacteria have been killed by antibiotic treatments could promote the re-colonisation of the surface by the same or other bacterial species, thereby causing infection recurrence or severe complications^10–12^. Consequently, removing the residual EPS matrix could be at least as crucial as killing bacteria in the management of biofilm infections. Additionally, due to the variability in the composition of *S. aureus* EPS matrix and the interaction between their multiple components, the strategies to disrupt the matrix should ideally target several constituents of the EPS matrix simultaneously^13^.

In *S. aureus* biofilms, poly-N-acetyl-β-(1–6)-glucosamine (PNAG; also known as polysaccharide intercellular adhesin PIA), proteins and extracellular DNA (eDNA) are broadly viewed as the main components of the EPS matrix^14^. PNAG helps biofilms form and enables bacteria to gain protection from the host immune system^15,16^. Adhesion to the surface and initiation of biofilm formation^17^ are also related to the expression of numerous proteins, such as cell wall-anchored (CWA) proteins, phenol soluble modulins (PSMs) and recycled cytoplasmic proteins found in both methicillin-susceptible *S. aureus* (MSSA) and methicillin-resistant *S. aureus* (MRSA) isolates^18^. The third main component, the extracellular DNA (eDNA) released from cells through controlled autolysis, is considered crucial for stabilising the structure of *S. aureus* biofilms^19^.

To avoid biofilm formation, previous studies have focused on the development of prophylactic therapies dedicated to limit the accumulation of EPS matrix components during bacterial growth, i.e. PNAG, biofilm-related proteins and eDNA^20–23^. However, although most of the proposed compounds were successful in preventing biofilm formation, they were unable to destroy mature biofilms^24^ and thus had only limited efficacy when tackling clinical infections. During the maturation of *S. aureus* biofilms, the amount of PNAG decreases while proteins gradually increase to finally play a critical role in the mature form^25^. Up to 24 types of CWA proteins have been described as being implied in biofilm formation^26,27^ so proteases could be good candidates even if their efficacy can be lower in vivo than in vitro due to rapid elimination and instability^28^. Several studies have already shown significant effects of proteases on the EPS matrix of *S. aureus* biofilm in vitro, but almost all of them were only tested or validated using a few reference strains or laboratory strains^29,30^ whereas the molecular composition of *S. aureus* biofilm can be strain-specific^31,32^. EPS-targeting substances should consequently be assessed on many isolates with different biofilm production capacities to select those with the broadest spectrum of activity.

We hypothesized that the destabilization of proteins and/or PNAG would both enhance the destruction of the EPS matrix and limit the adhesion by the surviving biofilm bacteria released during this process. However, the destruction of EPS matrix may not be systematically correlated to a decrease in embedded bacteria and thus, in chronic infections associated to a biofilm, the addition of antibiotics is systematically required. The antibiotics should at least preserve or enhance the destruction effect of non-antibiotics on the EPS matrix and better, in optimal combinations, increase the detachment or kill embedded biofilm bacteria.

To assess EPS-targeting combinations, we examined the destructive effects of eight non-antibiotic substances on the EPS matrix of biofilms formed by 24 representative isolates of *S. aureus*. By combining crystal violet staining, bacterial counts, and confocal laser-scanning microscopy (CLSM), we selected one protease and one non-enzymatic compound leading to significant degradation of the EPS matrix. The reduction of embedded biofilm bacteria was further tested by combining these substances with antibiotics.

## Results

### Broad screening and classification of isolates based on their biofilm production

A total of 73 clinical isolates of *S. aureus* from bovine mastitis, including 54 MSSA and 19 MRSA, plus one laboratory strain (SH1000) were tested for their capacity to form a biofilm. The 24 h-old biofilms, which were maximised in BHI plus 1% glucose, were stained with crystal violet (CV) (four independent experiments in quadruplicate). Three distinct classes of *S. aureus* isolates related to their biofilm biomass were obtained by K-means and agglomerative hierarchical clustering (AHC, Fig. S1).

Based on the profile plot and variance decomposition, the isolates were categorised into low (42%, 31/74), medium (20%, 15/74), and high (38%, 28/74) biofilm producers (Table S1). Specifically, 45% of MSSA (25/55) and 32% of MRSA (6/19) isolates were clustered as low producers, 16% of MSSA (9/55) and 32% of MRSA (6/19) isolates as medium producers, 38% of MSSA (21/55) and 37% of MRSA (7/19) isolates as high producers. Biofilm biomass largely varied among strains, but there was no significant divergence between MSSA and MRSA (Fig. S2a). Among these 74 isolates, two isolates were chosen from the medium producers (one MSSA and one MRSA) and two from the high producers (one MSSA and one MRSA) to rapidly screen the substances. Another larger subset of 24 representative isolates (14 MSSA and 10 MRSA) from the three clusters was selected to further characterise the most effective substances. The biofilm biomasses and the counts of biofilm bacteria for these 24 representative isolates are represented in Fig. S2b. The quantity of biofilm bacteria in the 24 representative isolates ranged from 4 to 7 log_10_ CFU/mL with weak linear correlation with the biofilm biomass assessed by CV staining (R^2^ = 0.486) (Fig. S2b).

### Selection of subtilisin A and calcium gluconate (CaG) out of eight non-antibiotic substances

**Figure 1:**
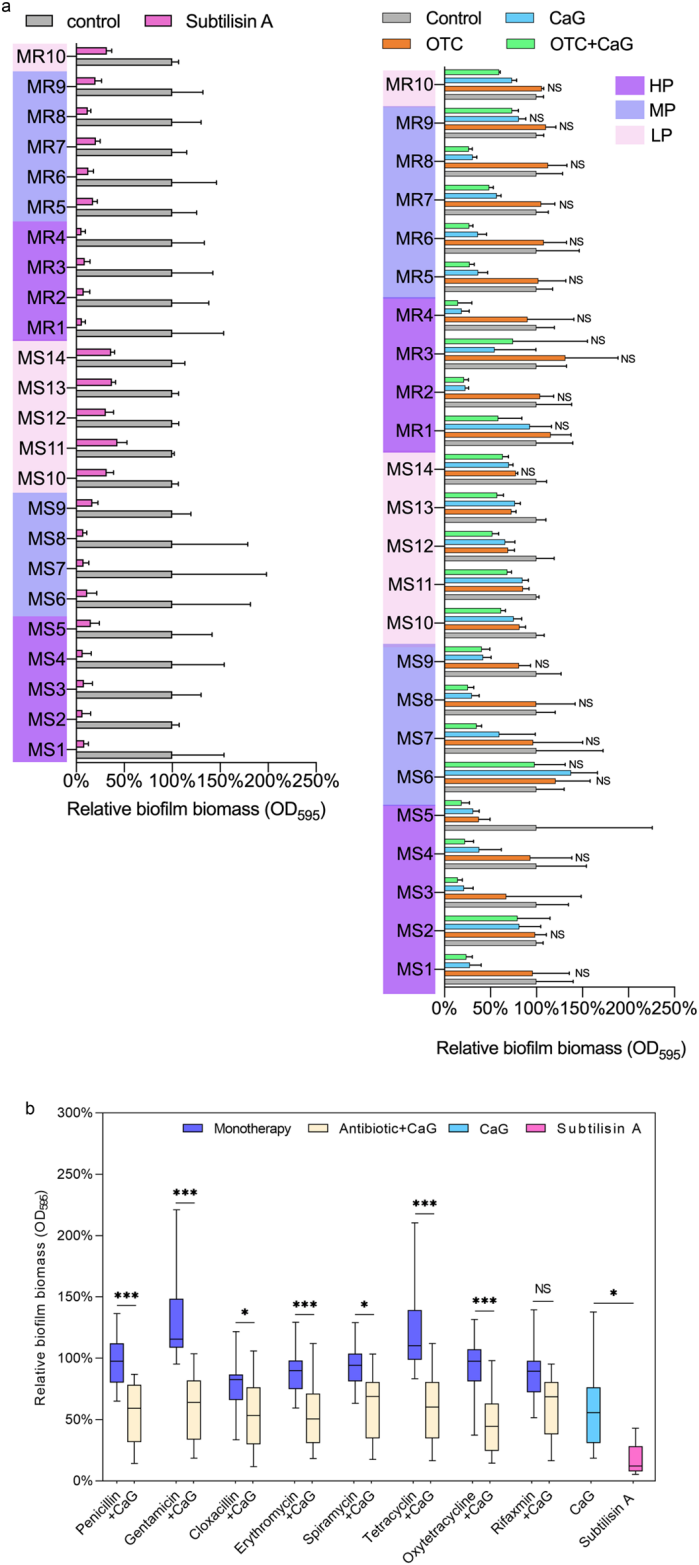
Reduction of *S. aureus* biofilm biomass by subtilisin A, Calcium gluconate (CaG), antibiotics alone and in combination. (**a**) Relative biomasses of 24 h-old biofilms of 24 isolates exposed to subtilisin A (0.01 U/mL), CaG (Ca^2+^ 1.25 mmol/L), OTC (10 μg/mL), and the combination of OTC and CaG for 24 h. The X-axes represent the percentage of biofilm biomass (OD_595_) with the control (biofilm formed by the non-treated isolate) set at 100%. High (HP), medium (MP) and low (LP) biofilm producers are highlighted with different colors. Statistical significance was determined by the Mann–Whitney U test. Unmarked: P < 0.05. NS: not significant. Error bars indicate the SD. (**b**) Relative biomass of 24 h-old biofilms exposed to eight antibiotics, subtilisin A (0.01 U/mL), CaG (Ca^2+^1.25 mmol/L) and to the combination of each antibiotic with CaG for 24 h. Results from the 24 isolates are represented as boxplots. Statistical significance was determined by Friedman's test with a post hoc application of Nemanyi. *P < 0.05 **P < 0.01. ***P < 0.001. NS: not significant. Other relevant P values are reported in Table S4. The Y-axis represents the percentage of OD_595_ values relative to the control group set at 100%. Data are the means of values from two independent experiments in triplicate (n = 6).

The effect of eight different non-antibiotic substances (Fig. S3 and Table S2) on total biofilm biomass was quantified on the small subset of four isolates in order to determine which ones had the potential to destroy the EPS matrix. The results showed a dramatic reduction in the biofilm biomass with proteinase K and subtilisin A, both of which resulted in lower OD_595_ values than with DNase I (Fig. S3a). Interestingly, calcium ions (1.25 mmol/L), which must be applied in combination with proteinase K to ensure its activity, also significantly reduced the total biofilm biomass (Fig. S4) and thus prevented the quantification of the effects of proteinase K alone. Consequently, subtilisin A was then selected and applied on the biofilms of the 24 representative isolates. This led to a reduction in total biofilm biomass for all 24 isolates (Fig. 1a). The lower relative efficacy observed for the low biofilm producers compared to the medium and high producers (Fig. S5) can be explained by the CV assay’s detection limit, which prevented the observation of a further reduction in the biofilm biomass of these low producers. It should be noted that the effect of a medium concentration of subtilisin A (0.01 U/mL) was similar to that of the high concentration (0.1 U/mL) (Fig. S3a).

### Control biofilm imaging

**Figure 2:**
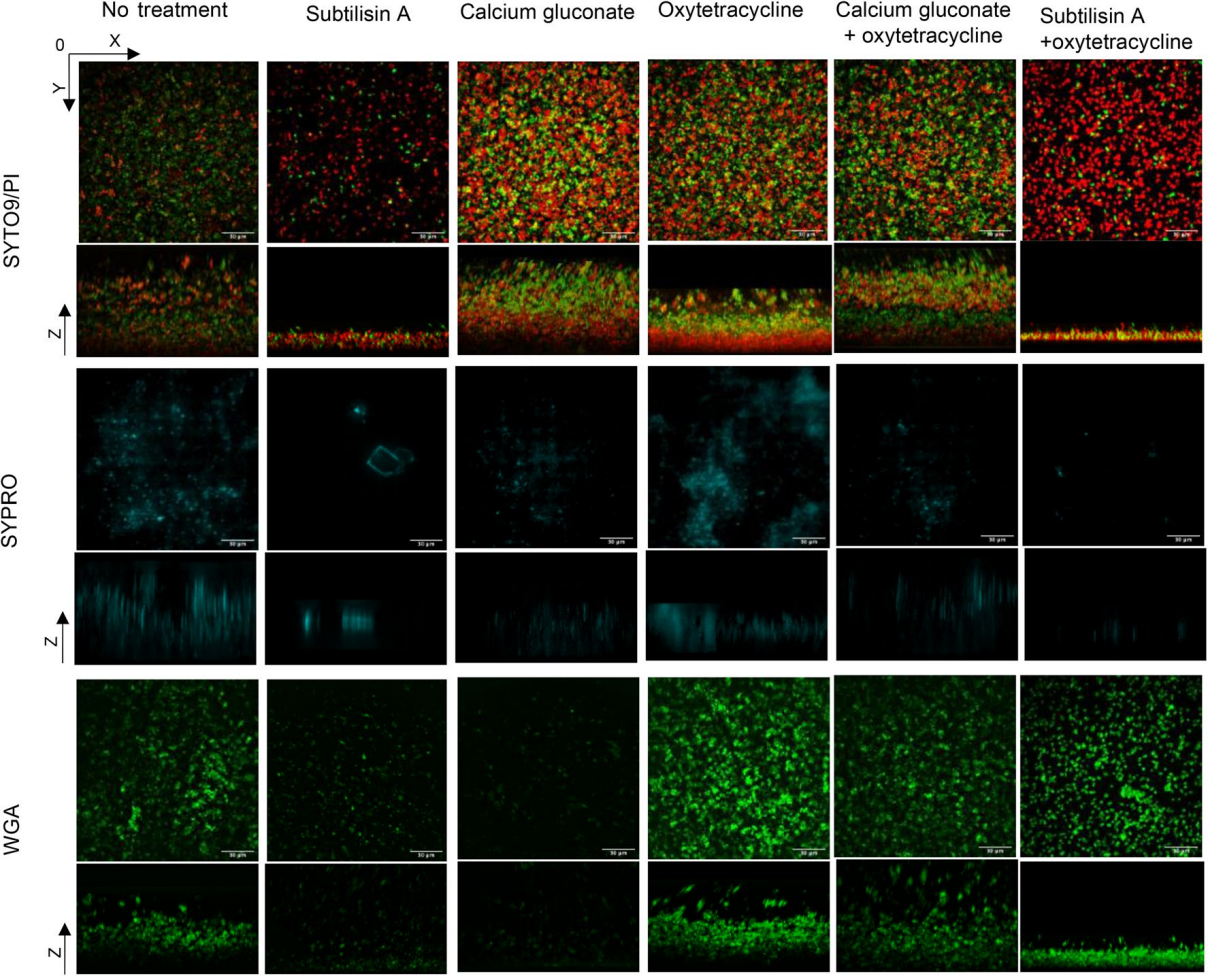
CLSM images of *S. aureus* biofilms exposed to the three substances tested. Representative 3D images of biofilms from MS3 isolate grown statically for 24 h and treated or untreated with subtilisin A (0.01 U/mL), CaG (Ca^2+^ 1.25 mmol/L), OTC (10 μg/mL) alone or in combination for 24 h. Biofilms were stained with the Syto9/PI, SYPRO Ruby biofilm matrix stain and wheat germ agglutinin (WGA). Scale bars, 30 μm.

The 3D images captured with CLSM after Syto9/PI staining showed that in the control *S. aureus* biofilm of the MS3 isolate (a high producer with an average OD_595_ value of 3.4 and average biofilm bacterial counts of 6.8 log_10_ CFU/mL), the PI stained (red) and the Syto9 stained (green) components were mixed in a thick biofilm with a higher density of the PI stained components at the bottom of the biofilm. The two additional staining procedures with WGA (wheat germ agglutinin) and SYPRO Ruby matrix stain indicated that the EPS matrix contained large amounts of poly-*N*-acetyl-β-(1–6)-glucosamine (PNAG) exopolysaccharide and proteins (Fig. 2).

### Characterisation of subtilisin A activity on biofilm

**Figure 3:**
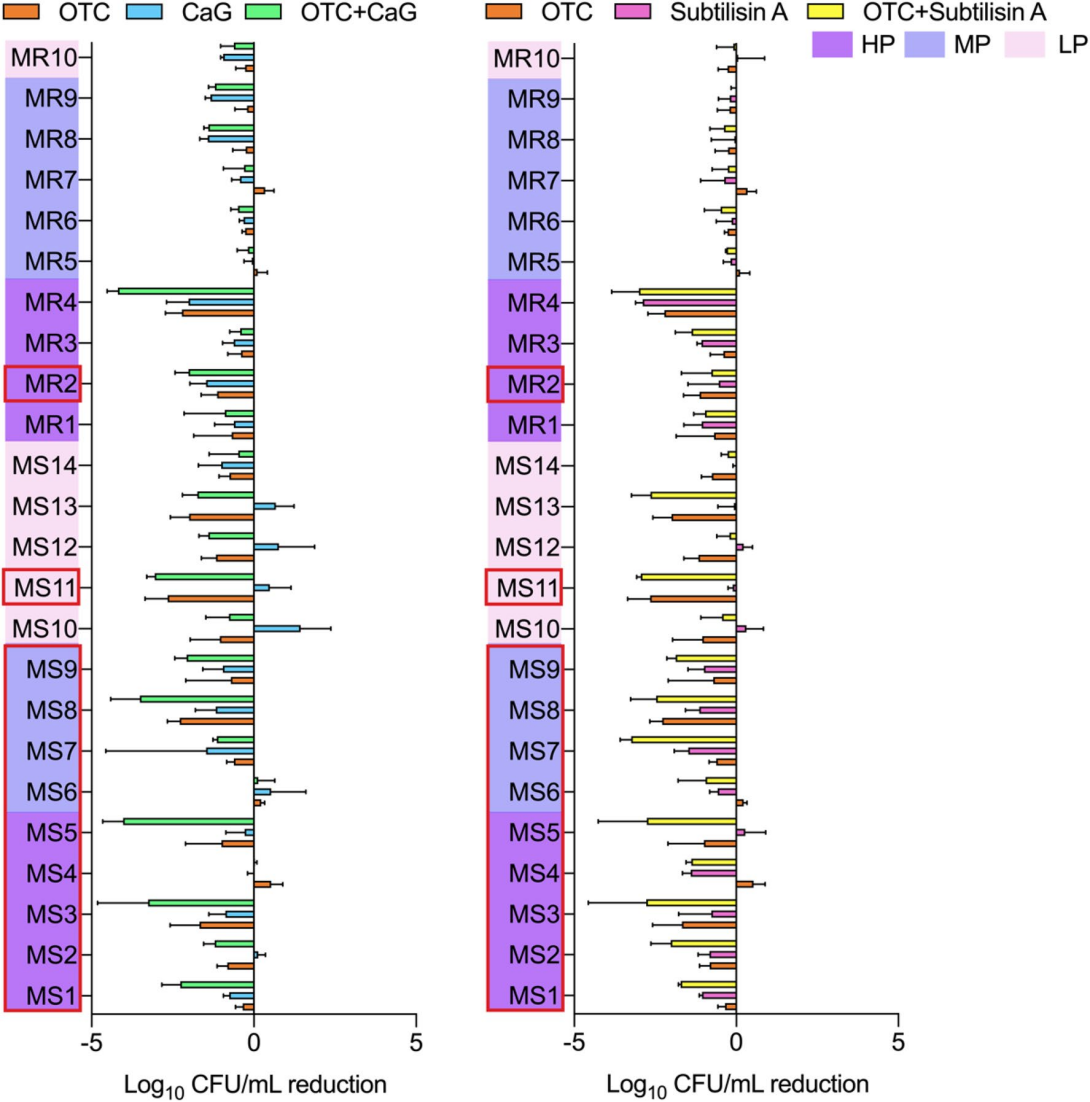
Reduction in the biofilm bacteria of 24 individual isolates by subtilisin A, CaG, and OTC alone or in combination compared to control. Data are the means of three independent experiments (n = 3). Error bars indicate the SD. The isolates for which the MBC of OTC is less than10 μg/mL are labelled in red frame. High (HP), medium (MP) and low (LP) biofilm producers are highlighted with different colors.

The MIC and MBC of subtilisin A were both higher than 0.05 U/mL for all 24 *S. aureus* isolates (Table S3), meaning that the tested concentration of 0.01 U/mL should neither kill bacteria nor inhibit bacterial growth. Treating the biofilm with subtilisin A for 24 h resulted in an approximate average reduction of 0.6 log_10_ CFU/mL in embedded biofilm bacteria that could be mainly explained by detachment (Fig. 3). The effect of subtilisin A was observed by CLSM on the MS3 isolate (Fig. 2), for which subtilisin A led to a relative biofilm biomass of 8% compared to the control set at 100% (Fig. 1a). Subtilisin A induced a dramatic decrease in biofilm thickness and the red and green stained components were limited to small individual pieces. The abundant proteins (SYPRO Ruby—blue stain) observed in the control were no longer visible. The PNAG (WGA-green stain) were rare and dispersed after subtilisin A monotherapy.

In parallel to the experiments on mature biofilm, subtilisin A (0.01 U/mL) added before incubation of bacteria for either 24 or 48 h significantly lowered the total biofilm biomass compared to the control for all the tested isolates, including high biofilm producers (Fig. S6).

### Characterisation of CaG activity on biofilm

During the preliminary selection of substances to test on four isolates, the addition of calcium ions led to an unexpected and dramatic reduction in the biofilm biomass for three out of four isolates at a concentration similar to the plasma concentration (Ca^2+^ 1.25 mmol/L) (Fig. S3a). CaG was therefore tested on the 24 representative isolates and induced a significant decline in biofilm biomass for 21/24 isolates (Fig. 1a). Interestingly, CaG led to a significant increase in biofilm biomass for the laboratory strain SH1000 (named here MS6) and had no significant effect on a further two isolates (MR1 and MR9). The values for the MIC (Ca^2+^ > 112.5 mmol/L) and MBC (Ca^2+^ > 112.5 mmol/L) of CaG for all isolates were far higher than the tested Ca^2+^ concentration of 1.25 mmol/L (Table S3). The average reduction of embedded biofilm bacteria after treatment with CaG was around 0.4 log_10_ CFU/mL (Fig. 3). CLSM performed on the MS3 isolate, for which CaG led to a relative biofilm biomass of 21% compared to the control set at 100% (Fig. 1a), showed that the live/dead staining in presence of CaG was quite similar to the control. The only difference was that PI-stained components appeared to be slightly more evenly distributed and aggregated at the bottom of the biofilm (Fig. 2). The marked difference between CaG and the control was observed for SYPRO Ruby and WGA staining, with much fewer stained components compared to the control even if the thickness of the stained layer (z-axis) after CaG remained far higher than after subtilisin A.

Like subtilisin A, CaG inhibited biofilm formation (Fig. S6) as shown by the marked reduction in biofilm biomass after incubation for 24 h (22/24 isolates) or 48 h (21/24 isolates).

### Selection of antibiotics to combine with subtilisin A and CaG

We initially assessed the ability of eight antibiotics alone at three different concentrations (Table S2) to reduce the biofilm biomass of the small subset of four isolates (Fig. S3b). Since the eight antibiotics failed to substantially reduce the biofilm biomass, only the highest concentrations were tested on the 24 representative isolates (Fig. 1b). Antibiotics alone led in the best cases to a very slight reduction in biofilm biomass (oxytetracycline (OTC), cloxacillin) and in the case of penicillin G, gentamicin and tetracycline, even led to a significant increase in biofilm biomass in respectively 5/24, 10/24 and 7/24 isolates compared to the control.

The very low OD values obtained after CV staining with subtilisin A alone were too close to the limit of detection to detect any enhanced effect by combining antibiotics with subtilisin A. Therefore, only the effects on biofilm biomass of a combination of antibiotics and CaG were explored by CV staining. The results, represented in Fig. 1b and Table S4, showed that the addition of antibiotics did not significantly reduce or enhance the efficacy of CaG on the biofilm biomass of the 24 isolates. Among all the combinations, OTC combined with CaG led to the lowest average for the relative biofilm biomass (46%) compared to the control set at 100% (Fig. 1b). Moreover, this combination significantly decreased the individual biofilm biomass for 22 out of 24 isolates compared to the control (Fig. 1a). This effect was independent of the MSSA or MRSA status, and did not depend on biofilm production of the isolates (Fig. S5). As for CaG alone, the only isolate whose biofilm biomass actually increased after exposure to the combination was the laboratory SH1000 strain (MS6, Fig. 1a).

**Figure 4:**
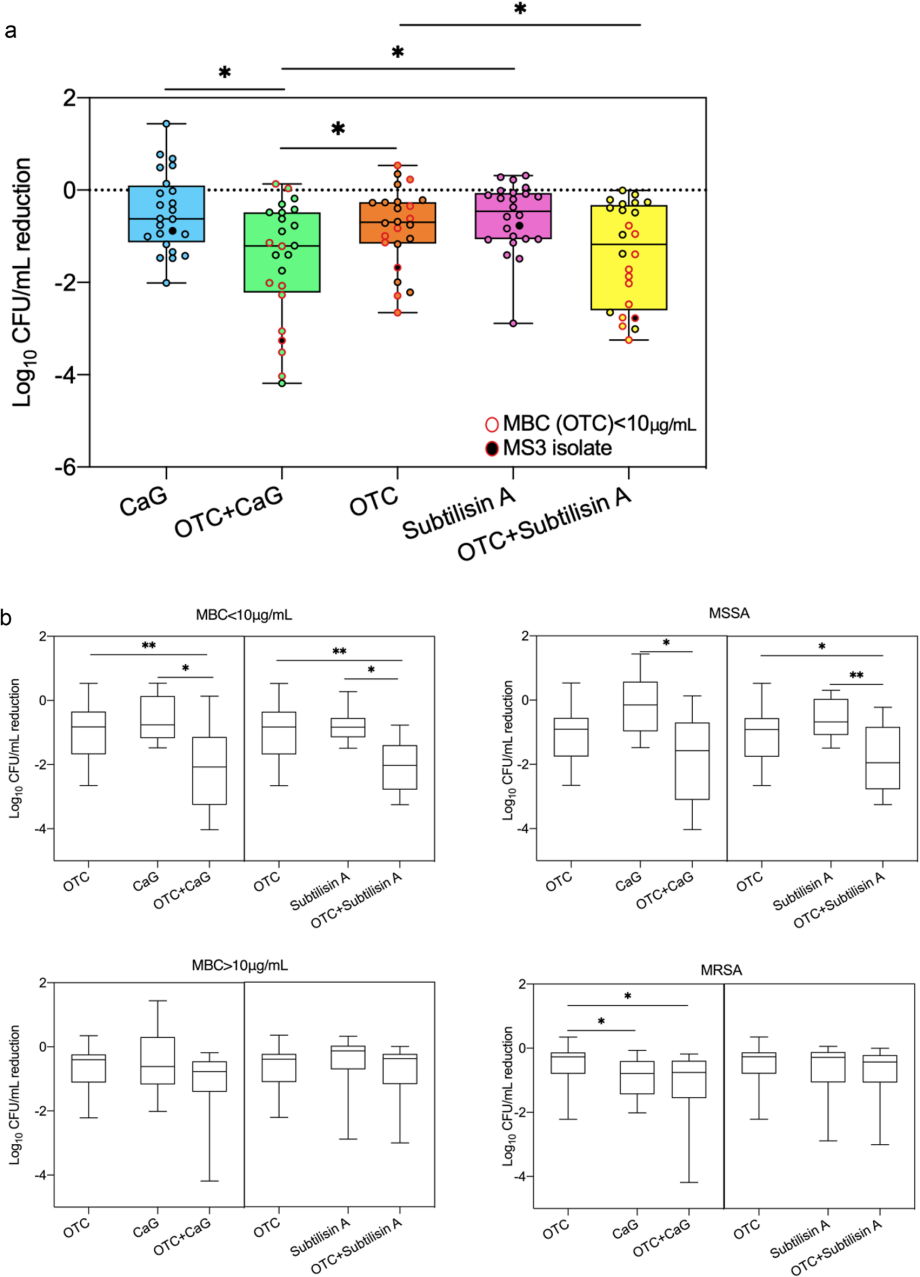
Reduction in *S. aureus* biofilm bacteria after exposure to subtilisin A, CaG, OTC alone and in combination. (**a**) Reduction in biofilm bacteria counts after exposure to OTC (10 μg/mL), subtilisin A (0.01U/mL), and Ca^2+^ (1.25 mmol/L) alone and in combination represented as box plots for the 24 isolates. Each isolate is represented by a circle. Statistical significance was determined by Friedman's test with a post hoc application of Nemanyi. *P < 0.05. (**b**) Reduction in biofilm bacteria counts after exposure to OTC (10 μg/mL), subtilisin A (0.01 U/mL), and Ca^2+^ (1.25 mmol/L) alone and in combination represented as separate box plots according to the MBC of OTC (MBC < 10 μg/mL or MBC > 10 μg/mL) and MSSA/MRSA status for the individual isolates. Statistical significance was determined by the Friedman's test with a post hoc application of Nemanyi. *P < 0.05, **P < 0.01. No mark: not significant. Data are the means of three independent experiments (n = 3).

The MIC of OTC for the 24 isolates ranged from 0.0625 to 0.25 μg/mL for MSSA and from 0.0125 to 128 μg/mL for MRSA (Table S3). The MBC ranged from 0.25 to 32 μg/mL for MSSA and from 4 to > 128 μg/mL for MRSA with 11/24 isolates having an MBC lower than 10 μg/mL (Table S3). The average reduction of embedded biofilm bacteria exposed to 10 µg/mL of OTC alone was only of 0.8 log_10_ CFU/mL (Fig. 4a) and was not correlated to the isolates’ MBC (Fig. 4b).

### Characterisation of the effects of the combination of subtilisin A or CaG with OTC on biofilm

Both combinations (OTC + CaG and OTC + subtilisin A) led to a significantly greater reduction in embedded biofilm bacteria by detachment or killing than OTC alone (Fig. 4a). OTC + CaG also showed a better effect than CaG alone, while OTC + subtilisin A showed no significant difference compared to subtilisin A alone (Fig. 4a). In order to identify factors explaining the differential effects of these two combinations on the representative isolates, we classified them depending on their susceptibility to methicillin (MSSA vs MRSA) and to OTC (MBC < 10 μg/mL vs > 10 μg/mL). The combination of OTC and subtilisin A had an additive effect (2 log_10_ CFU/mL average reduction) on the embedded biofilm bacteria from the MSSA isolates and from the isolates with MBC of OTC lower than 10 μg/mL (Fig. 4b). The additive effect of OTC combined with CaG was only observed for the isolates with MBC of OTC < 10 μg/mL (Fig. 4b). The reduction of embedded biofilm bacteria exceeded 3 log_10_ CFU/mL for 20%-25% of these isolates after the treatment by combinations (OTC + CaG and OTC + subtilisin A) (Figs. 3 and 4a).

After exposure to OTC alone, the PI staining of the MS3 isolate’s biofilm (MBC of OTC < 10 µg/mL) corresponding to dead cells or extracellular DNA or RNA increased and was concentrated at the bottom of the biofilm compared with the control group (Fig. 2). The image of biofilm staining by Syto9/PI after the combination of OTC and CaG was quite similar to the image with CaG alone. When OTC was combined with subtilisin A, the images were different from those of the control group, subtilisin A alone or OTC alone. After combined exposure to OTC and subtilisin A, the PI staining became fragmented and isolated. The thickness of the biofilm also decreased and was even thinner than biofilm after subtilisin A alone. In the presence of SYPRO Ruby stain (blue), the CLSM images of OTC alone and the control were similar, suggesting that OTC had no impact on the proteins. In combination, OTC did not impact the ability of CaG and subtilisin A to extensively reduce the protein content. In parallel, WGA staining showed that OTC increased the PNAG content of the biofilm matrix compared to the control group. The combination of OTC with subtilisin A or CaG led to higher PNAG content than subtilisin A or CaG alone, even though the combination with subtilisin A seemed to limit WGA staining to the wall of the bacteria (almost only surrounding single cells).

## Discussion

*S. aureus* infections associated with biofilms are difficult to eradicate because of the high tolerance of bacteria to antibacterial agents and to host immune defences. We selected and proved that subtilisin A (0.01 U/ml) and CaG (Ca^2+^ 1.25 mmol/L) inhibited the in vitro formation of the EPS matrix and, more importantly, destroyed the EPS matrix of several mature *S. aureus* biofilms, enabling for some isolates a significant reduction of embedded biofilm bacteria once combined with antibiotics.

In most studies related to *S. aureus* biofilms, assays were conducted on a few laboratory strains and especially on the NCTC 8325 lineage^33^. Although this is relevant for comparisons between related studies, it can lead to a selection of drugs with a narrow spectrum of activity since the main biofilm structural components could depend on the strains^32^. To better identify broad-spectrum drugs, we decided to select 24 out of 73 clinical isolates from bovine mastitis (plus one laboratory strain, *S. aureus* SH1000) with different biofilm biomass productions to test both the non-antibiotic and antibiotic substances.

By comparing biomasses (OD values after CV staining) and bacterial counts for the biofilm formed by 24 isolates after 24 h, we observed that there was a weak linear correlation between biomass and bacterial counts, suggesting that both methods should be implemented in parallel to obtain information on the effects of substances on the matrix and the bacteria. A decrease in the biomass would indicate a decrease in the biofilm matrix content, in embedded dead cells or adherent living biofilm bacteria whereas a decrease in bacterial counts would indicate bacterial detachment or killing within the biofilm.

Among the eight non-antibiotic substances tested, subtilisin A (a serine endopeptidase produced by *Bacillus subtilis*) significantly reduced the biofilm biomass. The lower efficacy of DNase I could result from the supplementation with glucose in the broth, which was shown to reduce the release of eDNA from cells^32^. Since proteinase K needs calcium for its activity, a control experiment was conducted using calcium alone and revealed that the activity of proteinase K could be partly explained by calcium ions. Therefore, proteinase K was excluded while calcium gluconate (CaG) was kept for further experiments. Subtilisins have been described as having a broad-spectrum activity on proteins^34^, and are already widely used to control biofilm in the food industry and to reduce water pollution ^35–37^. In our study, the reduction in biofilm biomasses by subtilisin A was not specific to either MSSA or MRSA. It did not depend on the different strains’ production of biofilm either, thus supporting broad-spectrum activity. The ability of subtilisin A to prevent biofilm formation was also confirmed. Compared to the reduction in biofilm biomass, subtilisin A reduced cultivable bacteria inside the biofilm only slightly, with an average reduction of about 0.6 log_10_ CFU/mL. This suggests that the decrease in biomass could be explained by degradation of the EPS matrix.

For the high biofilm producer isolate (MS3), CLSM demonstrated that, under control conditions, live biofilm cells were embedded in PI-stained components that can be dead cells, extracellular DNA or small parts of RNA^19,38,39^ gradually integrated in the biofilm matrix. The staining of PNAG and proteins also showed that MS3 control biofilm contained both components. The images suggested that subtilisin A destroyed the biofilm structure. A similar effect has previously been observed with high concentrations of ficin (1000 μg/ml), which is also a protease^40^. Furthermore, a significant decrease in biofilm thickness was observed with subtilisin A while there was little bacterial reduction (0.76 log_10_ CFU/ml) for the MS3 isolate’s biofilm, thus confirming that the EPS matrix was massively destroyed. In accordance with the protease activity of subtilisin A, no protein was stained in the EPS matrix but more surprisingly, the PNAG content was also greatly reduced. One explanation could be that, by degrading proteins, subtilisin A triggered the collapse of the entire biofilm structure. However, the ability of subtilisin A to also degrade PNAG and other biofilm-related components needs to be further assessed.

CaG also significantly reduced the biomass of the mature biofilm for 21/24 isolates. The tested calcium concentration of 1.25 mmol/L was consistent with the concentration of free calcium ions in mammals’ blood (strictly maintained between 1.1 and 1.3 mmol/L)^41,42^, and slightly lower than the concentration of free calcium ions in human plasma (2.2 to 2.7 mmol/L). This concentration is far below the MIC of calcium, so it should have had no bactericidal or inhibitory effects^43^ and suggested again that calcium can damage the formed EPS matrix. Like subtilisin A, CaG also prevented biofilm formation. Calcium ions mainly play a structural role in the maintenance of cell wall integrity (extracellular calcium-binding proteins)^43,44^. In 2004, Arrizubieta et al. ^45^ demonstrated that calcium ions at 10 mmol/L inhibited Bap-mediated *S. aureus* biofilm formation by binding to the identified EF-hand-like domains of the Bap protein, thus rendering proteins incompetent for biofilm formation and intercellular adhesion. Similarly, in our experiments, CaG at 1.25 mmol/L (Ca^2+^) inhibited biofilm formation in 22/24 isolates. Interestingly, the only isolate that grew after exposure to CaG was laboratory strain SH1000. CaG did not reduce the biomass of this strain’s mature biofilm but was still able to inhibit EPS matrix formation. It is unlikely that the inhibitory and destructive effects of CaG on the EPS matrix are due solely to the presence of the *bap* gene, as calcium can potentially bind to the EF-hand domain in other proteins. Abraham et al. ^46^ showed that calcium ions at 3.125 mmol/L disrupted established biofilms by binding to the Clf-B protein and a study by Lee et al.^47^ also demonstrated the inhibitory effect of calcium ions on the biofilm formation of an isolate from a human lesion. The CLSM images showed that the decrease in protein and PNAG content within the EPS matrix with CaG was similar to the decrease observed with subtilisin A, even though the biofilm remained far thicker with calcium than with subtilisin A. One hypothesis is that the proteins in the EPS matrix could be degraded by subtilisin A, whereas calcium ions could cause a conformational change in Bap that affects its ability to form biofilms^45^.

Since subtilisin A and CaG alone destroyed the matrix without extensively reducing embedded biofilm bacteria, we then investigated combining each of them with antibiotics. The first criterion for the antibiotic selection was that the addition of antibiotics should preserve or increase the destructive effect of subtilisin A and CaG on the EPS matrix. The eight antibiotics tested had very little effect on the biofilm biomass as previously demonstrated in many studies^48–50^. Even if not statistically significant, the greatest reduction in biomass obtained by combining CaG with an antibiotic was obtained with OTC, which was thus selected for further experiments. We observed through CLSM that OTC alone killed very few biofilm bacteria. Interestingly, OTC led in parallel to a significant increase in PNAG compared to the control. Similar observations were also reported with penicillin G, which increased PNAG while reducing the bacterial population and biofilm biomass^51^. The slight killing effect associated with the increase in PNAG in presence of OTC could explain why the global biofilm biomass between OTC treatment and the control was similar, and supports the use of CLSM to clarify drugs’ mechanism of action. Even if OTC can bind to calcium^52^, the combination of calcium and OTC led to a greater reduction in biofilm biomass than other combinations, and to a significant additive effect on the removal of embedded biofilm bacteria. The added calcium suppressed the enhanced effect of OTC on PNAG in the EPS matrix and the effect of the combination on proteins was similar to the effect of calcium alone. These effects on PNAG, proteins and bacteria were in accordance with the significant reduction in total biofilm biomass by a combination of OTC and calcium compared to the control. Similarly, the addition of subtilisin A inhibited or reduced the enhanced effect of OTC on PNAG as there were no more visible cell clumps after the combination of both. This might be explained by an increase in dead or detached bacteria or by extensive destruction of the matrix. The remarkable protein degradation of subtilisin A was preserved when used in combination with OTC and caused a significant decrease in total biofilm biomass and thickness. We further found that both combinations (OTC + subtilisin A and OTC + CaG) had a significant additive effect (killing or detachment) on the embedded biofilm bacteria for the 11/24 isolates having an MBC lower than the tested concentration of OTC (10 μg/mL). As MSSA are usually more susceptible to OTC^53,54^, we had more MSSA included in the group of MBC < 10 μg/mL. However, there was no significant additive effect of OTC + CaG on the embedded biofilm bacteria when the isolates were classified as MSSA vs MRSA groups. Villa et al*.*^55^ showed that the susceptibility of biofilm bacteria to ampicillin was enhanced when subtilisin A was used to prevent the biofilm formation of *Escherichia coli*. Another study showed that an engineered peptidoglycan hydrolase degrading the peptidoglycan structure of *S. aureus* could increase bacterial killing and biofilm eradication by gentamicin in animal models^56^.

In conclusion, by using several approaches to explore biofilm in parallel, we characterised the mode of action of compounds with anti-biofilm activity and selected efficient combinations. This study specifically demonstrated that subtilisin A or calcium can extensively disrupt the matrix of many *S. aureus* isolates of animal origin. The combination of subtilisin A or CaG with OTC produced an additive reduction of embedded biofilm bacteria for isolates highly susceptible to OTC (MBC < 10 μg/mL). This suggests that subtilisin A and CaG may reveal the activity of OTC on biofilm bacteria and that they could also probably be combined to other antibiotics depending on the strain specificity.

## Materials and methods

### Bacterial strains

A total of 73 clinical isolates of *S. aureus* were included in this study. All the isolates were collected in France through the Resapath network for the surveillance of resistance in veterinary medicine (https://resapath.anses.fr/). Susceptibility testing using disc diffusion according to the guidelines of the French Society for Microbiology (CA-SFM) showed that 54 isolates were methicillin-susceptible *S. aureus* (MSSA), and 19 isolates were methicillin-resistant *S. aureus* (MRSA). The presence of the *mecA* gene was assessed by PCR. We used *S. aureus* SH1000 as the positive control to ensure that the low biofilm production observed for some isolates was not caused by the experimental conditions.

### Biofilm formation

All the isolates were first cultured on Mueller Hinton Agar overnight at 37 °C. A few colonies were then diluted in sterile Brain Heart Infusion (BHI) broth plus 1% glucose to obtain a final suspension of 6 log_10_ CFU/mL and 180 μL of this suspension was added to each well of a 96-well polystyrene microplate (Thermofisher Nunc). Plates were incubated at 37 °C for 24 h to allow biofilm formation before adding substances, except for experiments carried out to assess the ability of subtilisin A and CaG to inhibit biofilm formation. In this case, the substances were added to the wells at the same time as bacteria.

### Crystal Violet (CV) assay (biomass)

After a further 24 h of incubation at 37 °C in the presence of substances or only BHI with 1% glucose (control), the microplates were turned upside down and tapped so that broth and most of the planktonic bacteria fell out onto the absorbent mat^57^. The biofilms were then rinsed twice with 200 μL of sterile phosphate-buffered saline per well. Two hundred microlitres of CV solution (Sigma-Aldrich, 0.05% w/v) was added to each well and left for 3 min at 25 °C. The excess dye was then rinsed with 200 μL of sterile PBS, the wells were air-dried naturally, then 200 μL of 96% (w/v) ethanol was added to each well to resolubilise the residual dye. The biofilm’s biomass was measured using a microplate reader (CLARIOstar Plus) with the value of absorbance set at 595 nm (OD_595_).

### Quantification of cultivable biofilm bacteria

After 24 h of incubation at 37 °C in the presence of substances or only BHI with 1% glucose (control), wells were rinsed twice with 200 μL of sterile PBS as above and the bacteria were then resuspended in 200 μL of sterile PBS. After 10 min of ultrasound at 40 Hz (Branson), the cultivable biofilm bacteria were counted by plating serial tenfold dilutions on tryptic soy agar plates^58^. The reduction in biofilm bacteria was calculated as the difference between counts after exposure to substances and counts in control wells.

### Classification of isolates depending on biofilm production and selection of subsets

The averages of four OD values obtained in quadruplicate for each of the 74 isolates without added substances were used to perform k-means clustering followed by agglomerative hierarchical clustering (AHC) in order to classify the isolates according to their biofilm biomass-forming capacity using XLSTAT (2019.4.2version). The results of this classification followed by the counting of live bacteria in control biofilms led to the selection of two representative subsets of 4 and 24 isolates out of the 74 strains in total in order to conduct assays with the different substances.

### Assessment of the efficacy of different substances

Proteinase K (Sigma-Aldrich, USA), subtilisin A (Sigma-Aldrich, USA), EDTA (Sigma-Aldrich, USA), phytosphingosine (Sigma-Aldrich, USA), lactoferrin (Sigma-Aldrich, USA), DNaseI (protease-free) (Roche Diagnostics, Germany), chlorhexidine digluconate (Intervet, France) and calcium gluconate (Lavoisier, France) were tested as non-antibiotic substances. The tested antibiotics were benzylpenicillin G, gentamicin, cloxacillin, spiramycin, erythromycin, tetracycline and oxytetracycline (OTC) purchased from Sigma-Aldrich (St.Louis, USA) and rifaximin obtained from TRC (Toronto, Canada). After 24 h of biofilm incubation, 20 µL of solutions containing the tested substances was added to each well. For the control wells, BHI with 1% glucose was added. After 24 h at 37 °C (except chlorhexidine digluconate, which had 2 min of treatment), the effect of treatment was measured by a CV assay and bacteria count. For the CV assay, three OD values in duplicate (n = 6) were obtained for each isolate and each condition. Triplicate bacteria counts (n = 3) were performed for each isolate under each tested condition. The preliminary experiments were conducted on four isolates and the effects of the substances with the highest efficacy were then assessed on 24 isolates.

The Minimum Inhibitory Concentration (MIC) of OTC, subtilisin A, and CaG was determined for the 24 isolates in triplicate by the microdilution method according to the CLSI^59^. The Minimum Bactericidal Concentration (MBC) was tested in triplicate as described in CLSI document M26-A^60^.

### Confocal laser scanning microscopy

CLSM was used to examine 24 h-old biofilm exposed or not to substances. The selected isolate was the MS3 isolate which was representative of high producers of biofilm biomass. The biofilms were formed on a 6-well polystyrene microplate and washed with sterile PBS (except for the wheat germ agglutinin stain, for which sterile water was used) as described above. The biofilms were stained with Syto9/PI (Live/Dead kit, Molecular Probes), FilmTracer SYPRO Ruby biofilm Matrix stain (Molecular Probes), and wheat germ agglutinin AlexaFluro 488 conjugate (Invitrogen). Images were acquired on a spinning disc from Perkin Elmer with the CSU-X1 scan head. The biofilms were observed using a 40× water immersion objective (Fluor 40×/0.8W, Nikon). The biofilm image stacks were obtained at 499 × 500 pixels (two HAMAMATSU C9100-13 EMCCD cameras) in three different areas of each surface analysed. 3D reconstructions were created by ImageJ software.

### Statistical analysis

Mann–Whitney U tests were performed to compare the biofilm biomasses (OD values) of each control and treated isolate, to compare MSSA and MRSA isolates, and to compare isolates with an MBC for OTC lower or higher than 10 μg/mL. A Kruskal–Wallis test with Dunn’s multiple comparison post-hoc analysis was used to determine whether there was a statistically significant difference in the biofilm biomasses produced by the different groups according to their biofilm-forming capacity. For multiple comparisons of OD values and counts measured after different treatments, statistical significance was determined by a Friedman test with post hoc application of Nemanyi. A *p* value < 0.05 was considered statistically significant.

## Supplementary Information

Supplementary Information
