# Supplementary material for: Destruction of Staphylococcus aureus biofilms by combining an antibiotic with subtilisin A or calcium gluconate
Source: Sci Rep. 2021 Mar 18;11:6225. doi: 10.1038/s41598-021-85722-4 (PMC7973569; doi:10.1038/s41598-021-85722-4)
Supplement: Supplementary file 1 — Supplementary Information [file 41598_2021_85722_MOESM1_ESM.pdf]

Destruction of *S. aureus* biofilms by combining an antibiotic with subtilisin A or calcium gluconate

JingJing Liu[1,2], Jean-Yves Madec[1], Alain Bousquet-Mélou[2], Marisa Haenni[1], Aude A Ferran[2]

[1]Untié Antibiorésistance et Virulence Bactériennes, Université de Lyon – ANSES laboratoire de Lyon, Lyon, France
[2]INTHERES, INRAE, ENVT, Université de Toulouse, France

FIGURES

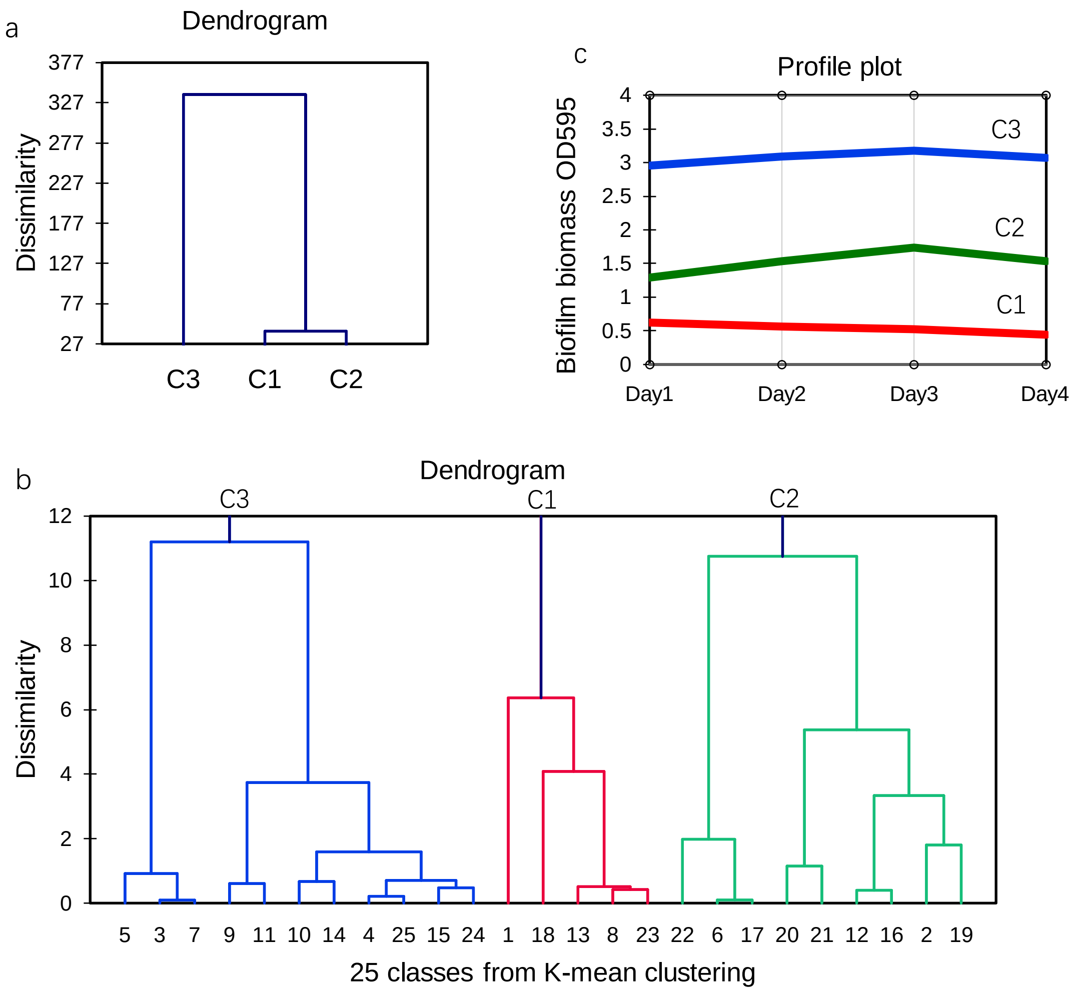

Figure. S1 Clustering of biofilm biomass producers. (a) The averages of the four OD values obtained each day for each isolate were first clustered into 25 classes by k-means. (b) The class centroids obtained were then analysed by agglomerative hierarchical clustering (AHC), which produced (c) three distinct clusters of S. aureus isolates related to their biofilm biomass. Four independent experiments in quadruplicate.

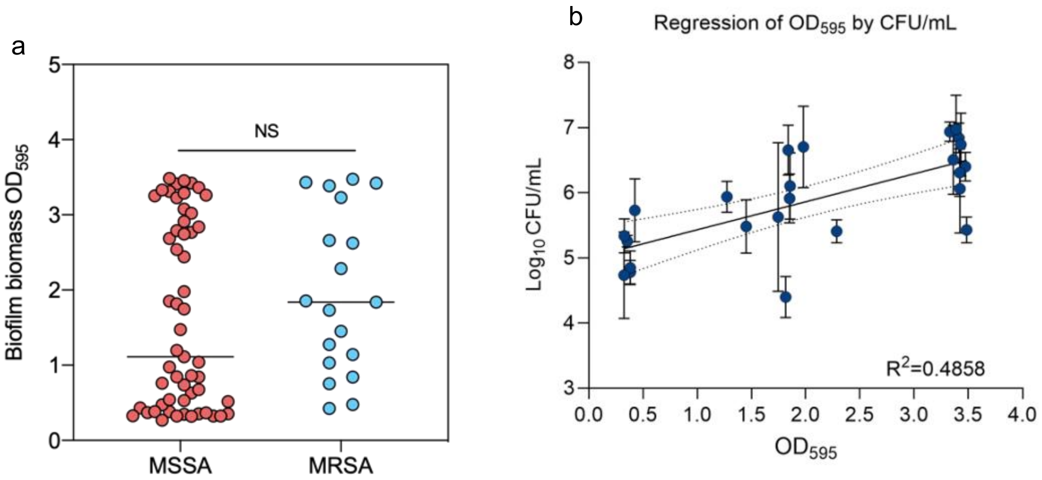

Figure S2. (a) The scattergrams of biofilm biomass production of MSSA and MRSA isolates. Data are the means of values from four independent experiments with four replicates. (b) Weak statistically significant fit for linear regression between biomass and biofilm bacteria counts. Biofilm biomass (OD_{595} values) and counts of biofilm bacteria (log_{10} CFU/mL) were obtained from 24 representative *S. aureus* isolates. OD values are the means of values obtained from four independent experiments with four replicates (n=16 for each isolate). The log_{10} CFU/mL data are the means of values obtained in at least three independent experiments with replicates (n>=6 for each isolate). Error bars indicate SD.

a. Non-antibiotic substances

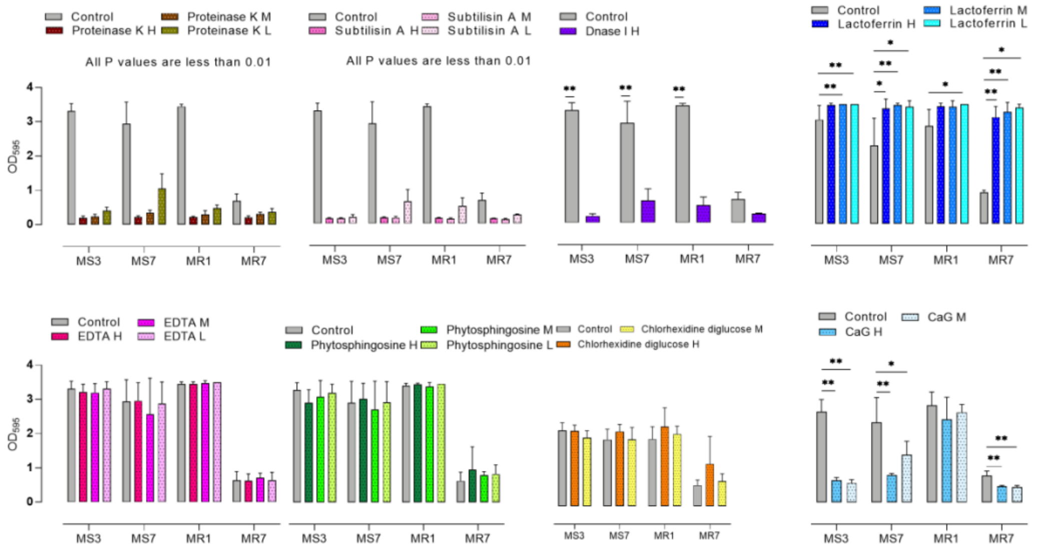

b. Antibiotic molecules

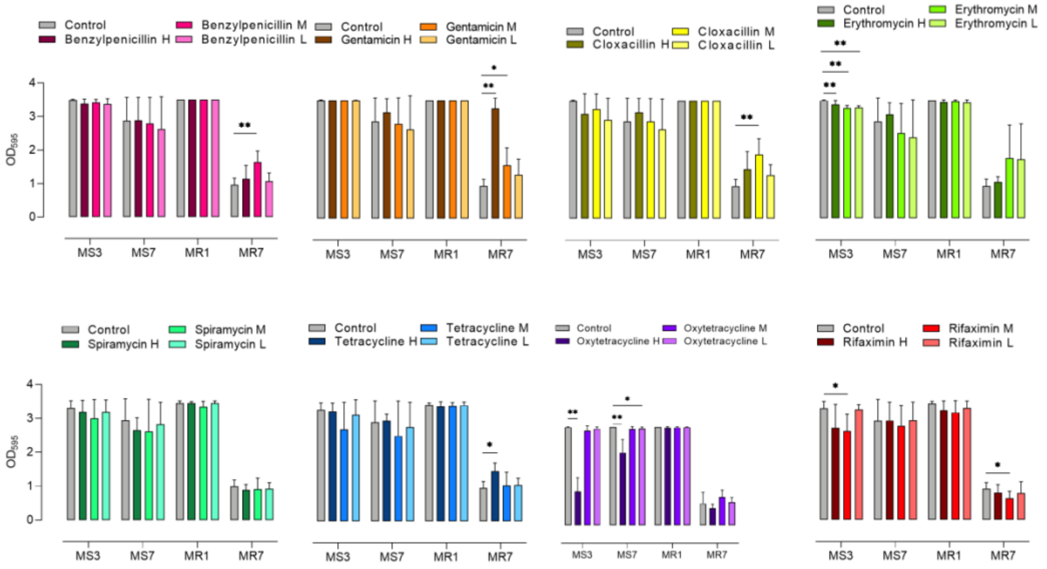

Figure S3. Preliminary selection of substances based on a reduction in biofilm biomass for two isolates (MS3 and MR1) from the high biomass producer group and two isolates (MS7 and MR7) from the medium biomass producer group out of 24 representative isolates. The destructive effect on biofilm biomass was measured (OD_{595} value) after exposure of biofilms to (a) eight non-antibiotic substances (proteinase K combined with Ca^{2+} 1.25mmol/L, subtilisin A, Dnase I, lactoferrin, EDTA, phytosphingosine, chlorhexidine digluconate, and calcium gluconate (CaG)) and (b) eight antibiotic molecules (benzyl-penicillin, gentamicin, cloxacillin, erythromycin, spiramycin, tetracycline, oxytetracycline (OTC), and rifaximin). The concentrations (H: high; M: medium; L: low) tested for each substance are reported in Table S2. Statistical significance compared to control was determined by the Mann-Whitney U test. *P<0.05.**P<0.01. ***P<0.001. Without a mark: not significant. Data are the means of values from two independent experiments in triplicate (n=6). Error bars indicate the SD.

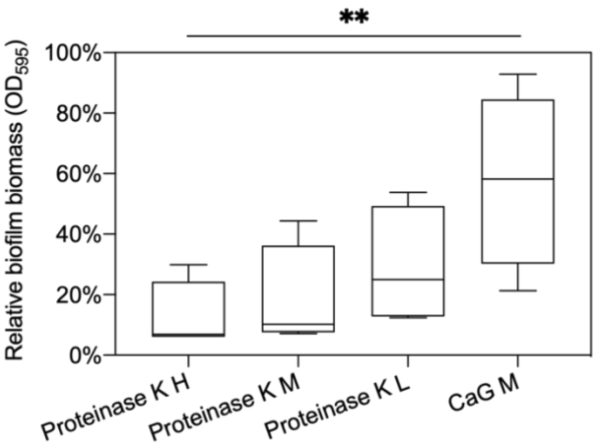

Figure S4. Reduction in biofilm biomass by CaG (1.25 mmol/L) alone or with proteinase K. Proteinase K was used with calcium chloride (Ca^{2+} 1.25mmol/L). H: high concentration; M: medium concentration; L: low concentration. Results from a preliminary selection (4 isolates) are represented as boxplots. Statistical significance between the different conditions was determined by Friedman's test with a post hoc application of Nemanyi. **P<0.01. Without a mark: not significant. The Y-axis represents the percentage of OD_{595} value relative to the control group. Data are the means of values from two independent experiments in triplicate (n=6). Error bars indicate the SD.

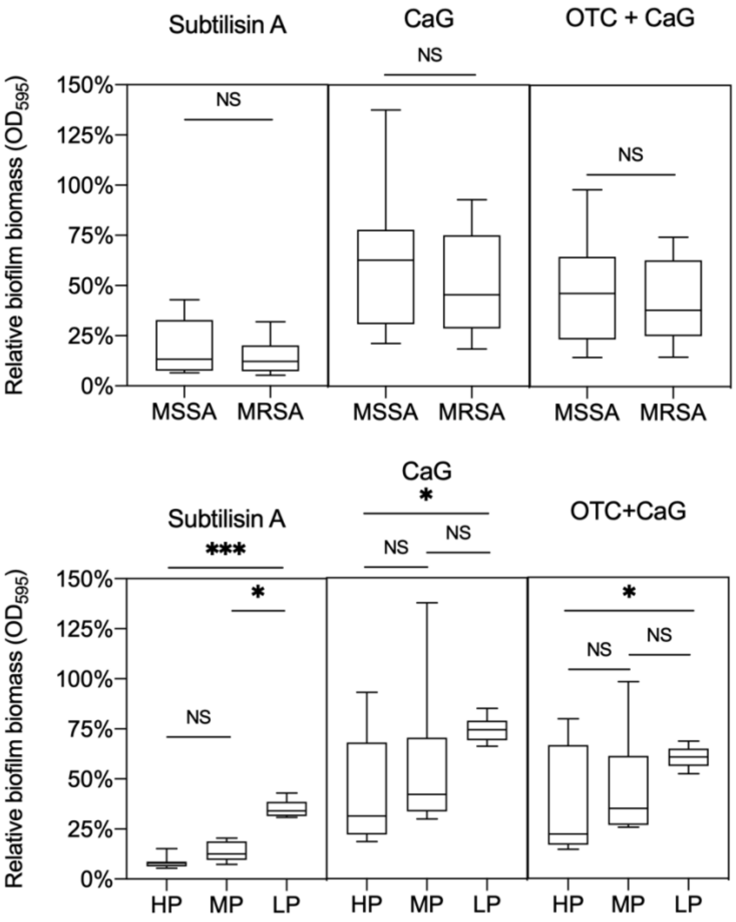

Figure S5. Reduction in biofilm biomass (24 isolates) after exposure to subtilisin A (0.01 U/mL), Ca^{2+} (1.25 mmol/L) or a combination of CaG and OTC (10 µg/mL) represented as separate box plots according to (a) MSSA and MRSA profiles or (b) to biofilm-forming capacity (high (HP), medium (MP), and low (LP) producer) for the individual isolates. Statistical significance between conditions was determined by the Mann-Whitney U test (for MSSA and MRSA) and Kruskal-Wallis test (for biofilm-forming capacity) respectively. *P<0.05.**P<0.01.***P<0.001. NS: not significant. Data are presented as box plots with values from two independent experiments in triplicate (n=6).

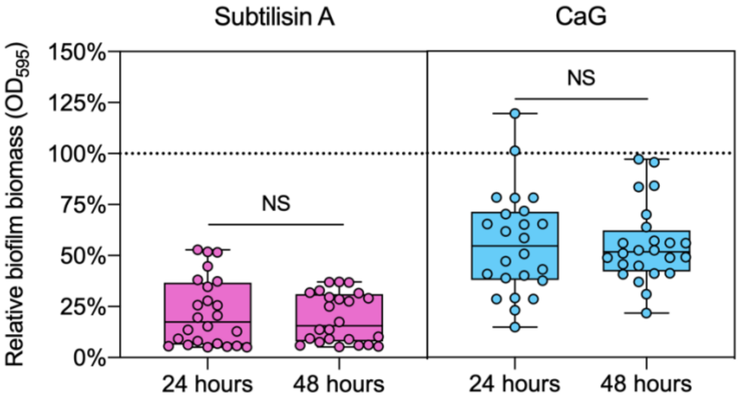

Figure S6. Inhibition of biofilm formation by subtilisin A and CaG. Relative biofilm biomasses from 24 representative isolates after addition of subtilisin A (0.01 U/mL) or CaG (Ca^{2+}1.25 mmol/L) at the onset of the biofilm. The inhibitory effects on the biofilm biomass were measured after incubation for 24 hours or 48 hours. Statistical significance was determined by the Mann-Whitney U test. NS: not significant. The Y-axis represents the percentage of OD_{595} value relative to the control group, itself set at 100%. Data are presented as box plots of two independent experiments in triplicate (n=6). Error bars indicate the SD.

TABLES

Table S1: Numbers and MSSA/MRSA profiles of the isolates according to their capacity to produce biofilm

Cluster of isolates	Total (74 isolates)		MSSA (55 isolates)		MRSA (19 isolates)	
	Percentage of isolates	OD_{595} range	Percentage of isolates	OD_{595} range	Percentage of isolates	OD_{595} range
Cluster 1: Low producer	42% (31/74)	1.1~0.3	45% (25/55)	0.9~0.3	32% (6/19)	1.1~0.4
Cluster 2: Medium producer	20% (15/74)	2.3~1.0	16% (9/55)	2.0~1.0	32% (6/19)	2.3~1.3
Cluster 3: High producer	38% (28/74)	3.5~2.4	38% (21/55)	3.5~2.4	37% (7/19)	3.5~2.6

Table S2: Tested concentrations for the eight non-antibiotic substances and eight antibiotics

Final concentration	Unit	High	Medium	Low
Proteinase K (with 1.25 mmol/L Ca^{2+})	µg/mL	100	10	1
Subtilisin A	U/mL	0.1	0.01	0.001
DNase I (Buffer)	U/mL	100	N/A	N/A
Lactoferrin	µg/mL	500	50	5
EDTA	µg/mL	10	1	0.1
Phytosphingosine	µg/mL	1	0.1	0.01
Chlorhexidine digluconate	% (w/v)	0.005	0.0005	N/A
Calcium gluconate	Ca^{2+} mmol/L	12.5	1.25	N/A
Benzyl-penicillin	µg/mL	100	10	1
Gentamicin	µg/mL	20	2	0.2
Cloxacillin	µg/mL	20	2	0.2
Erythromycin	µg/mL	200	20	2
Spiramycin	µg/mL	50	5	0.5
Tetracycline	µg/mL	10	1	0.1
Oxytetracycline	µg/mL	10	1	0.1
Rifaximin	µg/mL	5	0.5	0.05

Table S3: Minimum Inhibitory Concentration (MIC) and Minimum Bactericidal Concentration (MBC) of OTC, subtilisin A, and CaG for the 24 isolates. The isolates for which the MBC of OTC is less than10 μg/mL are labelled in red.

Isolate No.	Oxytetracycline (μg/mL)		Subtilisin A (U/mL)		Calcium gluconate (mmol/L)	
	MIC	MBC	MIC	MBC	MIC	MBC
MS1	0.125	0.25	>1.5	>1.5	>112.5	>112.5
MS2	0.125	8	>1.5	>1.5	>112.5	>112.5
MS3	0.125	4	>1.5	>1.5	>112.5	>112.5
MS4	0.125	8	>1.5	>1.5	>112.5	>112.5
MS5	0.125	8	>1.5	>1.5	>112.5	>112.5
MS6	0.125	0.5	>1.5	>1.5	>112.5	>112.5
MS7	0.25	4	>1.5	>1.5	>112.5	>112.5
MS8	0.125	2	>1.5	>1.5	>112.5	>112.5
MS9	0.125	2	>1.5	>1.5	>112.5	>112.5
MS10	0.0625	16	0.1875	0.1875	>112.5	>112.5
MS11	0.125	4	0.09375	0.09375	>112.5	>112.5
MS12	0.125	16	0.1875	0.1875	>112.5	>112.5
MS13	0.125	16	0.046875	0.046875	>112.5	>112.5
MS14	0.125	32	0.09375	0.09375	>112.5	>112.5
MR1	16	128	>1.5	>1.5	>112.5	>112.5
MR2	0.125	4	0.375	0.375	>112.5	>112.5
MR3	0.125	64	0.1875	0.1875	>112.5	>112.5
MR4	0.125	16	>1.5	>1.5	>112.5	>112.5
MR5	8	32	>1.5	>1.5	>112.5	>112.5
MR6	16	>128	>1.5	>1.5	>112.5	>112.5
MR7	128	>128	>1.5	>1.5	>112.5	>112.5
MR8	16	128	>1.5	>1.5	>112.5	>112.5
MR9	16	128	0.1875	0.1875	>112.5	>112.5
MR10	128	>128	>1.5	>1.5	>112.5	>112.5

Table S4: Statistical significance of Fig.2b. The table only shows statistically significant p-values and study-relevant p-values.

	PENG	GEN	CLOX	ERY	SPIR	TC	RIFX	OTC	CaG	Sub A
PENG +CaG	P <0.001								1.00	0.11
GEN +CaG		P <0.001							1.00	P <0.001
CLOX +CaG			P <0.05						1.00	0.28
ERY +CaG				P <0.001					1.00	0.40
SPIR +CaG					P <0.05				1.00	P<0.01
TC +CaG						P <0.001			1.00	P<0.05
RIFX +CaG							0.38		1.00	P <0.001
OTC +CaG								P <0.001	0.64	1.00
CaG	P <0.001	P <0.001	0.19	P<0.01	P <0.001	P <0.0001	P <0.05	P<0.01	1.00	<0.05
SubA	P <0.001	P <0.001	P <0.001	P <0.001	P <0.001	P <0.001	P <0.001	P <0.001	P<0.05	1.00

Antibiotics: PENG, benzyl-penicillin; CLOX, cloxacillin; GEN, gentamicin; ERY, erythromycin; SPIR, spiramycin; TC, tetracycline; RIFX, rifaximin; OTC, oxytetracycline.
